# The F-Box Protein CG5003 Regulates Axon Pruning and the Integrity of the *Drosophila* Mushroom Body

**DOI:** 10.3389/fnmol.2021.634784

**Published:** 2021-02-25

**Authors:** Mengying Yang, Yige Guo, Shuran Wang, Changyan Chen, Yung-Heng Chang, Margaret Su-chun Ho

**Affiliations:** ^1^School of Life Science and Technology, ShanghaiTech University, Shanghai, China; ^2^Institute of Neuroscience, State Key Laboratory of Neuroscience, Shanghai Institutes for Biological Sciences, Chinese Academy of Sciences, Shanghai, China; ^3^University of Chinese Academy of Sciences, Beijing, China; ^4^Institute of Intervention Vessel, Shanghai Tenth People’s Hospital, Shanghai Key Laboratory of Signaling and Diseases Research, School of Life Science and Technology, Tongji University, Shanghai, China; ^5^Department of Anesthesiology, Stony Brook School of Medicine, New York, NY, United States

**Keywords:** F-box, axon pruning, mushroom body, *Drosophila*, synaptic structure

## Abstract

Protein homeostasis serves as an important step in regulating diverse cellular processes underlying the function and development of the nervous system. In particular, the ubiquitination proteasome system (UPS), a universal pathway mediating protein degradation, contributes to the development of numerous synaptic structures, including the *Drosophila* olfactory-associative learning center mushroom body (MB), thereby affecting associated function. Here, we describe the function of a newly characterized *Drosophila* F-box protein CG5003, an adaptor for the RING-domain type E3 ligase (SCF complex), in MB development. Lacking CG5003 ubiquitously causes MB γ axon pruning defects and selective *CG5003* expression in pan-neurons leads to both γ axon and α/β lobe abnormalities. Interestingly, change in *CG5003* expression in MB neurons does not cause any abnormalities in axons, suggesting that CG5003 functions in cells extrinsic to MB to regulate its development. Mass spectrum analysis indicates that silencing *CG5003* expression in all neurons affects expression levels of proteins in the cell and structural morphogenesis, transcription regulator activity, and catalytic activity. Our findings reinforce the importance of UPS and identify a new factor in regulating neuronal development as exemplified by the synaptic structure MB.

## Introduction

Diverse behavior outputs rely on compartmentalized brain structures that function in a circuitry fashion. *Drosophila* mushroom body (MB) is the main olfactory-associative learning center in the adult brain and composed of three types of Kenyon cells (KCs) derived from four neuroblasts, each of them sequentially generates the γ,α′/β′, and α/β neurons (Ito et al., 1997; Lee et al., 1999; Noveen et al., 2000). By late larval stage (L3, 3rd instar), γ axons bifurcate into dorsal and medial lobes, both are completely pruned by 18 h after puparium formation (18 h APF), then re-projected to form the medial γ lobes in adults. α′/β′ neurons begin also in the larval stage to develop with axons projecting along a peduncle tract anteriorly, then bifurcates into dorsal α′ and medial β′ lobes. Likewise, MB α/β neurons then develop into dorsal α and medial β lobes at the beginning of puparium formation. These developmental and remodeling events make MB a great system to analyze intrinsic or extrinsic mechanisms regulating neuronal development.

The ubiquitination proteasome system (UPS) is a widely used mechanism to control protein turnover (Dikic, 2017; Pohl and Dikic, 2019). The UPS degradation machinery comprises a major enzymatic cascade that targets and covalently links the ubiquitin (Ub) chains to specific substrates. After the E1 activating enzyme utilizes ATP to form a high-energy thioester bond with Ub, the activated Ub is transferred to the E2 conjugating enzyme. The E3 ligase, either HECT or Cullin-based RING-type, recognizes specific substrates and catalyzes Ub-substrate conjugation from E2. Ultimately, the ubiquitinated substrates are sent for destruction by the 26S proteasome. The S phase kinase-associated protein 1 (SKP1)–cullin 1 (CUL1)–F-box protein (SCF) complex, a better-studied multi-subunit RING-type E3 ligase, provides the substrate specificity *via* the adaptor F-box protein (Ho et al., 2006, 2008). Substrates targeted for ubiquitination are often phosphorylated and interact with the substrate-binding domain of F-box protein (like WD repeats or leucine-rich repeats LRR).

Previous studies have shown that UPS regulates MB development (Watts et al., 2003; Zhu et al., 2005; Shin and DiAntonio, 2011; Wong et al., 2013; Meltzer et al., 2019). For instance, the E3 ligase Highwire is involved in MB axon guidance (Shin and DiAntonio, 2011), whereas Cul-1 and Cul-3 have been reported to regulate MB axon pruning and regrowth (Zhu et al., 2005; Wong et al., 2013), all in a cell-autonomous fashion. Here we report a newly characterized *Drosophila* F-box protein CG5003. CG5003 contains an F-box motif and interacts with Cul-1. Lack of CG5003 in the mutant background causes pruning defects of γ axons, indicating that CG5003 contributes to MB neuron remodeling. Also, selective CG5003 expression in pan-neurons, but not MB neurons, glia, nor DA neurons, causes both unpruned γ axons and thinned α/β lobes. Finally, mass spectrum analysis revealed possible CG5003 downstream effectors. These results suggest that CG5003 functions extrinsically to regulate MB development. Our findings identify a new factor in the UPS pathway that contributes to MB development.

## Materials and Methods

### Fly Strains and Genetics

Flies were maintained on standard fly food at 25°C with 70% humidity. All fly crosses were carried out at 25°C with standard laboratory conditions unless noted otherwise. All strains were obtained from Bloomington Stock Center, the Vienna *Drosophila* RNAi Center (VDRC), or as gifts from colleagues. Fly microinjection was conducted by the *Drosophila* Core Facility, Institute of Biochemistry and Cell Biology, Chinese Academy of Sciences.

### Immunohistochemistry and Western Blot Analysis

Whole-mount *Drosophila* adult brains were first dissected and fixed with 4% paraformaldehyde for 45 min. Samples were washed with PBT (PBS + 0.3% TX-100) three times and dissected further to remove additional debris in PBS solution. Clean and fixed brains were blocked in PBT solution with 5% Normal Donkey Serum (NDS) and subsequently probed with primary and secondary antibodies in solution with 5% NDS at 4°C overnight. Primary antibodies used in this study included: mouse anti-FasII-1D4 (1:50, DSHB) and mouse anti-Trio (1:50, DSHB), and anti-CG5003 (1:100). Secondary antibodies used from Jackson Lab included: rabbit anti-HRP-TRITC (1:500), donkey anti-mouse-Cy5 (1:200), donkey anti-rabbit Cy3 (1:200), and donkey anti-rabbit Cy5 (1:200).

For western blot analysis, fly tissue samples from adult heads were collected and lysed in lysis buffer. Protein extracts were then subjected to SDS-PAGE gel using antibodies against CG5003 and β-Actin.

### Confocal Microscopy and Statistical Analysis

Images of brains at different developmental stages were acquired by merging a serial Z-stack of average 35–40 sections, each of 0.35–0.5 μm thickness, using the Nikon A1 confocal microscope with 40× or 60× objective. Depending on the desired regions, the whole brains were positioned so that they can be scanned anteriorly to posteriorly (top to bottom). Approximately 40 sections were scanned and merged for visualizing the anterior MB lobes. The acquired MBs labeled by GFP or antibodies such as α-FasII were analyzed and quantified for the lobe defects. Average 10–15 brains (20–30 α/β lobes) were analyzed. The exact N number for each genotype is indicated in all Figures. Data were shown mean ± SEM. P-values of significance (indicated with asterisks, *p < 0.05, **p < 0.01, ***p < 0.001) were calculated using one-way ANOVA with Bonferroni multiple comparison test among three groups or above. Prism and SPSS software were used to complete the statistical analysis.

## Results

### *CG5003* Mutant Exhibits MB γ Axon Pruning Defects

**Figure 1:**
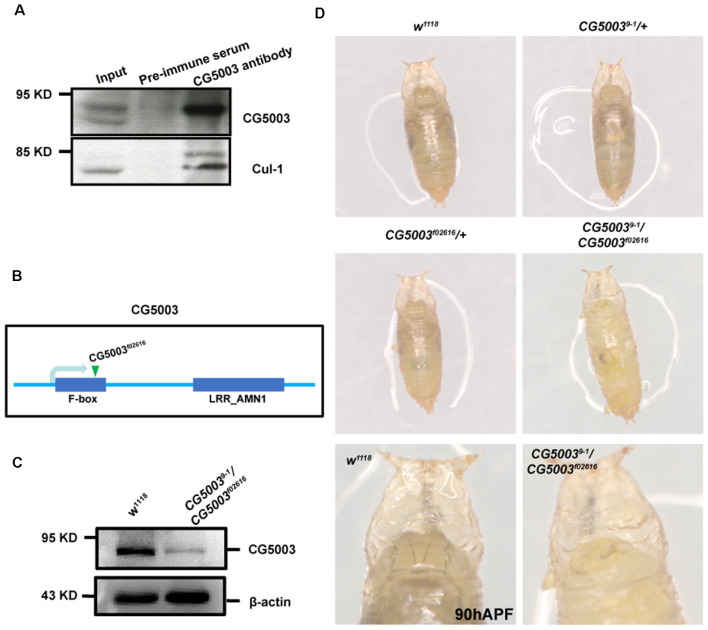
*9-1/f02616* is a *CG5003* mutant fly line. **(A)** Representative western blot images for CG5003 and Cul-1 interaction. Note that endogenous CG5003 proteins are in the same eluates with Cul-1, as pulled down by the anti-CG5003 antibodies. **(B)** Schematic diagram on CG5003 gene region. Note that *f02616* is a p-element insertion line located at the second exon. **(C)** Representative western blot images for analyzing CG5003 protein levels in the *9-1/f02616* mutants. Note a decrease in the CG5003 protein levels in the trans-heterozygotes. **(D)** Representative images of fly pupae with the indicated genotypes at 90 h after puparium formation (APF). Note that white-eye *9-1/f02616* mutant pupae exhibit head eversion normally in a similar time scale with the control.

To investigate CG5003 function in MB development, we first verified if CG5003 is a component of the SCF complex. Co-IPpull-downs demonstrated that CG5003 interacts with Cul-1 (Figure 1A), suggesting that CG5003 is an adaptor protein for the SCF complex. Next, a p-element insertion fly line *f02616* with the p-element inserted at the second exon of the gene region was examined (Flybase, Figure 1B). This line exhibits pupal lethality, indicating the possibility that *CG5003* expression levels are affected by the insertion. An EMS screen was then conducted to isolate additional alleles of *CG5003* for experimental purposes. Among all lines, line 9-1 was found pupal lethal, and trans-heterozygotes of 9-1 over f02616 (*9-1/f02616*) also caused pupal lethality. These results indicate that flies of line 9-1 fail to complement *f02616* and might carry a point mutation in *CG5003*. To further support the notion, western blot analysis was done to examine CG5003 protein levels in these flies. Interestingly, CG5003 protein levels were drastically reduced in *9-1/f02616* mutant flies, indicating that these trans-heterozygotes are suitable for examining the consequence of lacking CG5003 (Figure 1C).

**Figure 2:**
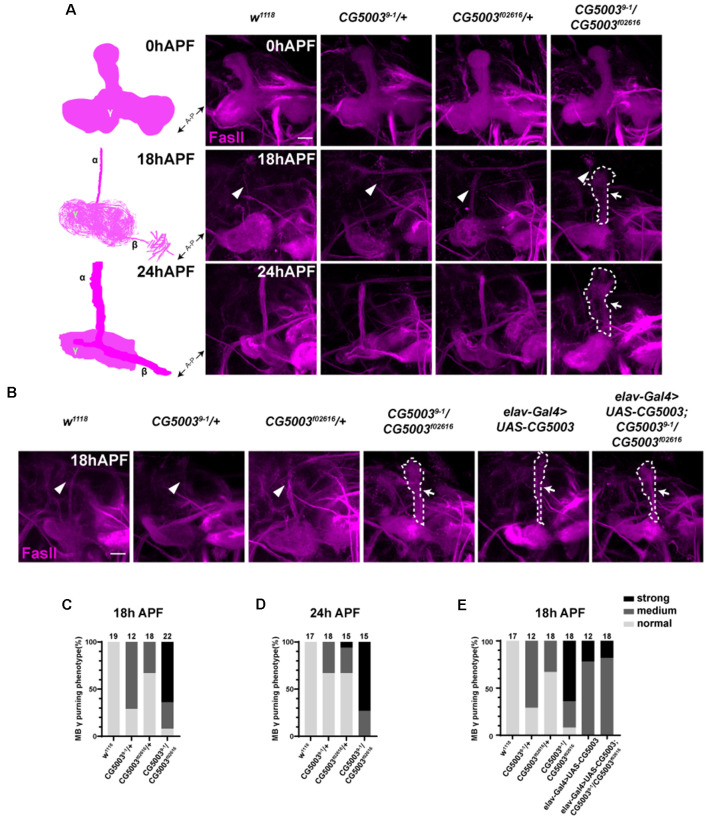
*CG5003* mutants exhibit mushroom body (MB) γ axon pruning defects. **(A–E)** Representative confocal images **(A,B)** and quantifications **(C–E)** of MB γ lobe formation of control and *CG5003* mutant fly brains at different developmental stages: 0 h APF, 18 h APF, and 24 h APF. Note that FasII-positive γ lobes are left unpruned at 18 h APF and 24 h APF in *CG5003* mutant MBs. *elav* driven *CG5003* expression partially rescued the mutant pruning defects. White arrows and dashed lines encircle the area of unpruned γ lobes. White arrowheads indicate the vertical α lobes. The *N* number of brains dissected and quantified for each genotype is indicated in the figure. Scale bar: 50 μm.

Taking advantage of *9-1/f02616* mutant flies, we first examined the developmental progress of these mutants. The head eversion occurs normally in *9-1/f02616* mutant pupae, suggesting that flies lacking CG5003 develop in a similar time scale as the wild-type flies (Figure 1D). Next, MB morphologies of *9-1/f02616* mutants at 3rd instar larvae, 18 h After Puparium Formation (APF), and 24 h APF were analyzed using the anti-FasII antibodies, a marker that stains α/β lobes strongly and γ lobes weakly (Crittenden et al., 1998). By 18 h APF, the γ axons were completely pruned in the wild-type MB. Whereas no significant difference of FasII-positive γ lobes across different genotypes was observed at 0 h APF, vertical γ lobes were present and left unpruned in *9-1/f02616* mutants at 18 h APF and 24 h APF (arrows and dashed areas in Figure 2A). Statistical analysis indicated that a significantly higher portion of unpruned γ lobes was present in the *9-1/f02616* mutants (Figures 2C,D). Given that the head eversion occurs normally, it is likely that the overall animal development is normal until the lethal pupal stage. However, due to the presence of these unpruned γ lobes, we were not able to discern potential α/β lobes. Thus, we cannot rule out the possibility that a delay in MB development as shown by the possible absence of α/β lobes at this stage occurs. To demonstrate that CG5003 expression affects axon pruning, CG5003 is re-introduced into the mutant background by expressing *CG5003* under the control of the pan-neuronal *elav-Gal4* driver. Interestingly, pan-neuronal *CG5003* expression partially rescued the mutant γ pruning defect (Figures 2B,E). Altogether, these results suggest that CG5003 is involved in MB γ axon pruning.

### Pan-Neuronal *CG5003* Expression Causes MB γ Axon Pruning Defects

**Figure 3:**
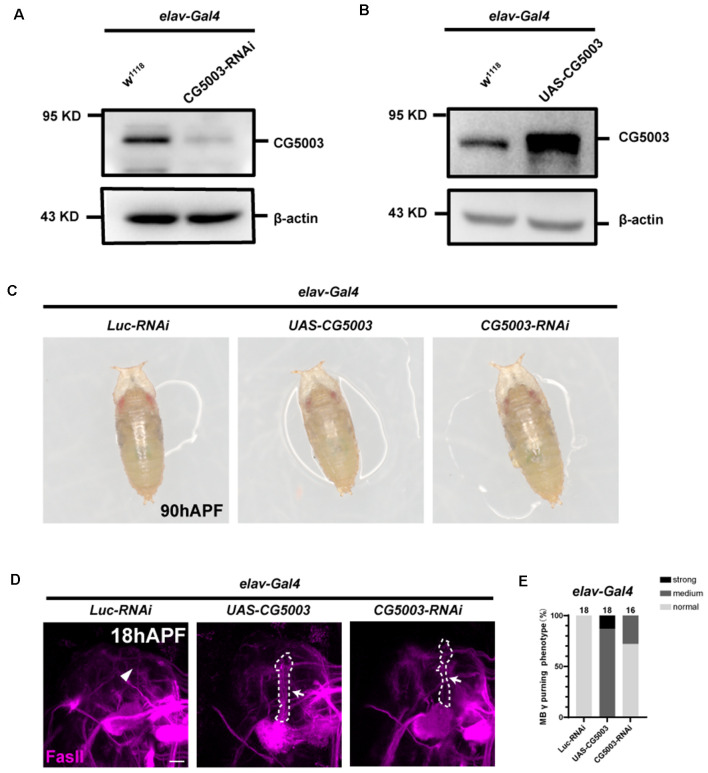
Pan-neuronal CG5003 expression causes MB γ axon pruning defects. **(A,B)** Western blot analysis on the protein extracts collected from fly pupae with the indicated genotypes. Note that CG5003 protein levels increase or decrease when *CG5003* or *CG5003-RNAi* are expressed, respectively. β-actin serves as an internal control. **(C)** Representative images of fly pupae with the indicated genotypes at 90 h APF. Note that red-eye *elav>CG5003* or *CG5003-RNAi* pupae exhibit head eversion normally in a similar time scale with the control. **(D,E)** Representative confocal images **(D)** and quantifications **(E)** of MB γ lobe formation of control, *CG5003*, and *CG5003-RNAi* fly pupae at 18 h APF. Note that FasII-positive γ lobes are left unpruned in MBs expressing *CG5003* or *CG5003-RNAi*. White arrows and dashed lines encircle the area of unpruned γ lobes. White arrowheads indicate the vertical α lobes. The *N* number of brains dissected and quantified for each genotype is indicated in the figure. Scale bar: 50 μm.

Based on the rescue results, we next examined whether pan-neuronal *CG5003* expression alone causes any defects in γ axon pruning. Transgenic flies carrying the RNAi targeting CG5003 (*CG5003-RNAi*, VDRC#26679) or *CG5003* were expressed using *elav-Gal4*. Efficiencies of these transgenes were validated by western blot analysis, revealing a corresponding reduction or increase in neuronal CG5003 protein levels (*elav>CG5003-RNAi or CG5003*, Figures 3A,B). These flies develop as the head eversion occurs normally (Figure 3C). As we examined the MB morphologies in 18 h APF, γ axons in a small portion of flies with pan-neuronal *CG5003-RNAi* expression were left unpruned, whereas the ones in flies with pan-neuronal *CG5003* expression were largely uneliminated and distorted (Figures 3D,E). Manipulation of *CG5003* expression using other Gal4 s, such as *C739-Gal4* (expresses in α/β neurons, Supplementary Figures 1A,D), 201Y-Gal4 (expresses in γ neurons, Supplementary Figures 1B,E), or TH-Gal4 (expresses in dopaminergic DA neurons, Supplementary Figures 1C,F), did not cause significant γ axon pruning defects. These results indicate that CG5003 likely functions in cells other than MB or DA neurons to regulate MB γ axon pruning.

### Pan-Neuronal *CG5003* Expression Causes MB α/β Lobe Defects

**Figure 4:**
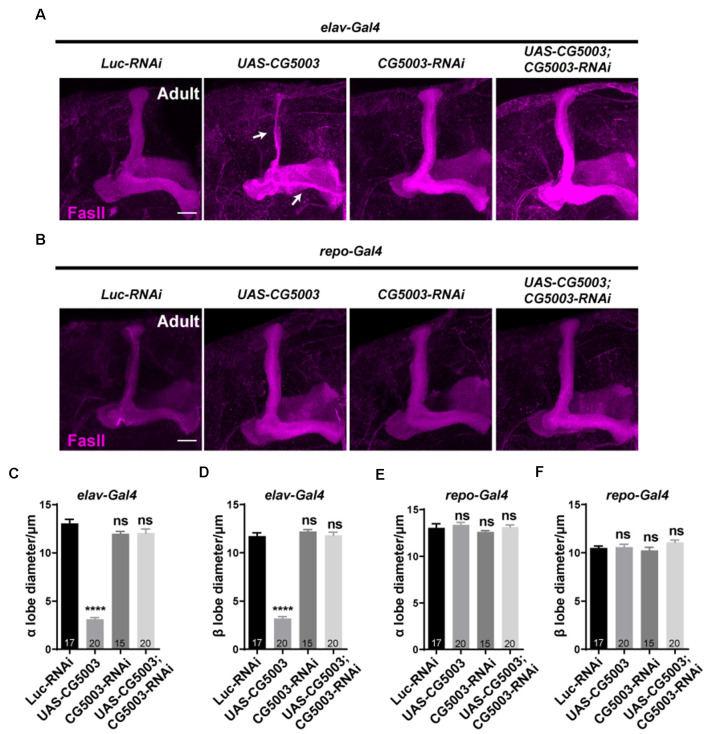
Pan-neuronal CG5003 expression causes MB α/β lobe defects. **(A–F)** Representative confocal images **(A,B)** and quantifications **(C–F)** of MB α/β lobe formation of control and experimental fly brains at the adult stage. Note that FasII-positive α/β lobes are severely disrupted when *CG5003* is expressed in all neurons. White arrows indicate the thinned α/β lobes. The *N* number of brains dissected and quantified for each genotype is indicated in the figure. Scale bar: 50 μm. ns, not significant; ****p < 0.000001.

In addition to γ axon pruning, we also investigated different stages of MB development such as α/β lobe formation in a later timeline. Interestingly, silencing *CG5003* expression in all neurons or glia did not cause significant distortion in FasII-positive MB α/β and γ lobes of 3-day-old adult flies (Figures 4A,C,D). Neuronal overexpression of *CG5003*, however, causes significant thinnings of α/β lobes, indicating that too much neuronal *CG5003* disturbs the proper development of MB α/β lobes (Figures 4A,C,D). On the other hand, MB α/β lobes remain normal upon either upregulating or downregulating *CG5003* expression in glia, suggesting that glial CG5003 does not play a significant role in regulating MB α/β lobe integrity (Figures 4B,E,F). Taken together, these results indicate that pan-neuronal *CG5003* expression regulates MB α/β lobe development in addition to γ axon pruning.

### Selective *CG5003* Expression in Subtypes of MB Neurons Does Not Affect MB α/β Lobe Integrity

**Figure 5:**
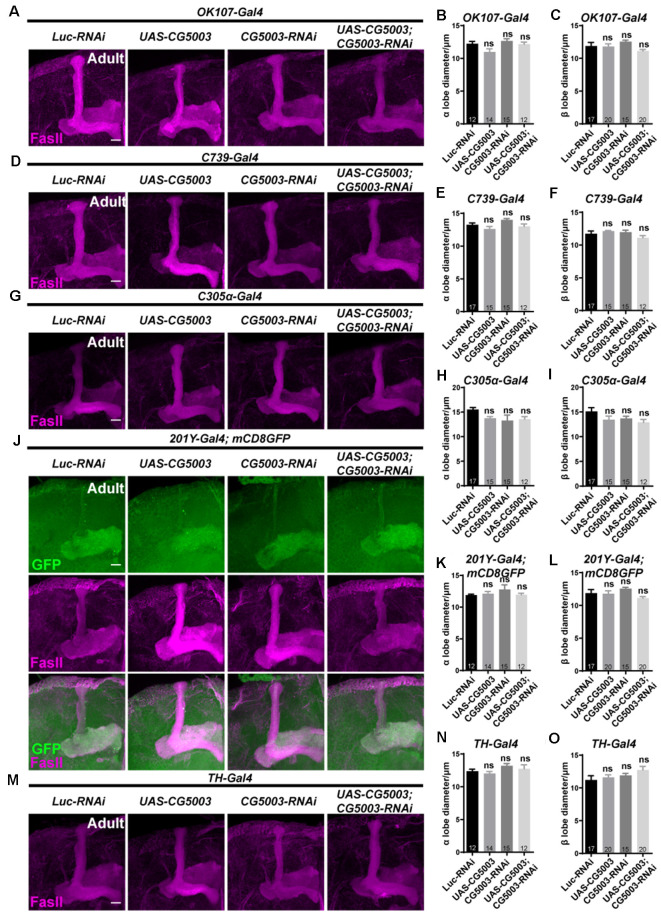
α/β lobes remain normal when altering *CG5003* expression in all or subsets of MB neurons. **(A–O)** Representative confocal images **(A–M)** and quantifications **(B,C,E,F, H,I, K,L, N,O)** of MB α/β lobe formation of control and experimental fly brains at the adult stage. Note that FasII-positive α/β lobes remain intact when *CG5003* or *CG5003-RNAi* is expressed in all MB (*OK-107-Gal4*), α/β (*C739-Gal4*), α’/β’ (*C305 α-Gal4*), γ (*201Y-Gal4*), or DA (*TH-Gal4*) neurons. The *N* number of brains dissected and quantified for each genotype is indicated in the figure. Scale bar: 50 μm. ns, not significant.

To further investigate whether CG5003 functions in a cell type-specific manner, transgenic *CG5003-RNAi* or *CG5003* was expressed under the control of different MB Gal4s that target all or subsets of MB neurons. Interestingly, no significant distortion in MB α/β and γ lobes of 3-day-old adult flies was observed when expressing *CG5003-RNAi* or *CG5003* using *OK107-Gal4* or *mb247-Gal4*, suggesting that the defects caused by *elav-Gal4* driven *CG5003* expression do not depend on MB neurons (Figures 5A–C; Supplementary Figures 2A,C,D). Furthermore, expressing *CG5003-RNAi* or *CG5003* using *C739-Gal4*, *C305α-Gal4* (targeting α’/α’ neurons), *201Y-Gal4*, or *TH-Gal4* did not cause significant distortion of MB α/β, α’/β’, and γ lobes, indicating that CG5003 does not act intrinsically in MB or DA neurons (Figures 5D–O; Supplementary Figures 2B,E,F). Taken together, these results implicate that CG5003 likely functions in cells other than MB or DA neurons to regulate MB development.

### Mass Spectrum Analysis Reveals Possible CG5003 Downstream Effector Proteins

**Figure 6:**
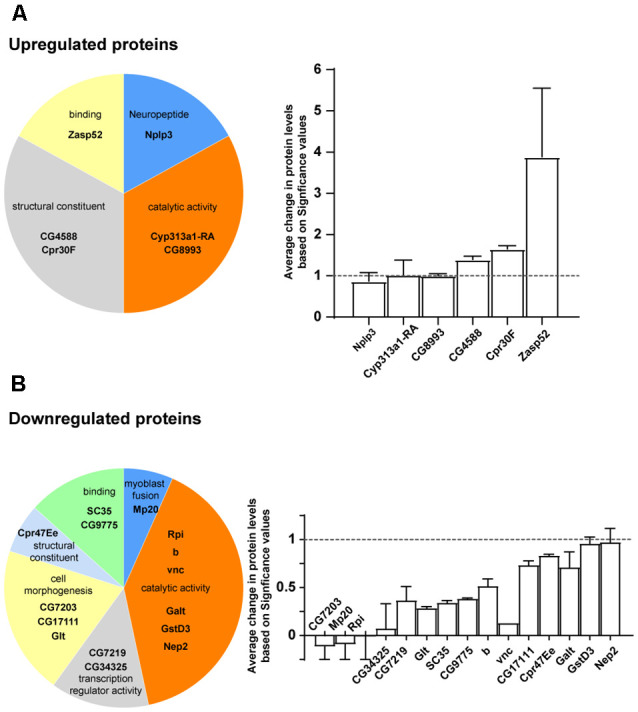
Mass spectrum analysis of CG5003 downstream effector proteins. **(A,B)** Representative proteins identified in mass spectrum analysis comparing samples from control and *elav>CG5003-RNAi* animals. The significance values are calculated based on protocols described previously (Li et al., 2013). Upregulated **(A)** and downregulated **(B)** proteins in different categories are shown. Protein annotations are indicated in each category.

To gain insights into the mechanism of CG5003-mediated MB development, mass spectrum analysis was performed using samples from control and *elav>CG5003-RNAi* animals. A total of approximately 4,000 proteins that exhibit differential expression levels between two samples were identified and categorized following the protocol described previously (Li et al., 2013). Approximately 20 out of these proteins were identified with the highest values in the difference of expression levels were shown in Figure 6. Interestingly, several novel genes including uncharacterized CG genes were identified. Moreover, a greater number of proteins involved in catalytic and transcription regulatory activity and fewer number of proteins involved in the structural morphogenesis were found to be affected by downregulating *CG5003* expression in all neurons. Vice versa, upregulated CG5003 levels affect mainly the protein expression in the structural morphogenesis levels. Taken together, these results provide insights on possible CG5003 downstream effectors regulating MB development.

## Discussion

UPS is commonly recognized as an important pathway regulating protein homeostasis using controlling protein degradation. Due to its prevalent roles, it is conceivable that UPS regulates the function and development of the nervous system. Our findings identify a new factor in the pathway, an adaptor F-box protein that regulates the development of MB. Lacking *CG5003* in all tissues causes MB γ axon pruning defects, whereas overexpressing *CG5003* in pan-neurons, but not MB nor DA neurons, leads to both γ axon pruning and α/β lobe thinning defects. These results demonstrate that CG5003 likely functions in cells other than MB or DA neurons in regulating MB development, further reinforcing the importance of UPS in neuronal development.

Previous studies have indicated that lacking the expression of the ubiquitin-activating enzyme (E1) or proteasome subunits in MB block γ axon pruning, suggesting that UPS is required for this process (Watts et al., 2003). It has also been shown that the E3 ligase Highwire regulates axon guidance of α/β neurons in a non-cell-autonomous fashion (Shin and DiAntonio, 2011). These findings all indicate an important requirement for UPS in the MB development. Interestingly, by analyzing the endogenous *CG5003* expression using a *CG5003* promoter-driven GFP fly line, it was found predominantly expressed in neuronal nuclei near the MB calyces, with some MB cell bodies expressing CG5003 within the calyces (Supplementary Figure 3). This expression pattern implicates that: first, CG5003 might function non-cell-autonomously in regulating MB development as its expression is predominantly seen outside of the MB calyces; second, a nuclear expression of *CG5003* might help explain its control over various transcription factor activity, potential CG5003 downstream effectors as revealed by the mass spectrum analysis.

Our findings identify a new F-box protein CG5003 that regulates MB development in two aspects: axon pruning and axon integrity. These regulations are likely from other extrinsic neurons to MB neurons. Intriguingly, the lack of *CG5003* causes similar pruning defects as pan-neuronal *CG5003* expression. It is possible that the induced *CG5003* expression results in an artificially higher level of CG5003 that causes a dominant-negative effect on pruning. Furthermore, the unpruned γ lobes were not detected in the adult stage, suggesting that other complementary mechanisms exist later in the MB development timeline to ensure pruning occurs and developmental events progress properly.

Since the change in *CG5003* expression affects both axon pruning and axon integrity, it is likely that CG5003 regulates a master step upstream of MB axon development, for instance, MB neuron differentiation. By identifying possible downstream substrates of CG5003 using mass spectrum analysis, the detailed mechanism of how UPS regulates MB development will be unraveled. Some of these identified proteins may be expressed in MB and regulated by CG5003. Future work will be required to investigate this unique aspect of UPS-mediated neuronal development.

## Data Availability Statement

The original contributions presented in the study are included in the article/Supplementary Material, further inquiries can be directed to the corresponding author.

## Author Contributions

MY and MH conceived and designed the study. MY, YG, SW, CC, and YC performed the experiments. MY and MH analyzed the data and wrote the article. All authors read and approved the manuscript.

## Conflict of Interest

The authors declare that the research was conducted in the absence of any commercial or financial relationships that could be construed as a potential conflict of interest.
