# Supplementary material for: The F-Box Protein CG5003 Regulates Axon Pruning and the Integrity of the Drosophila Mushroom Body
Source: Front Mol Neurosci. 2021 Feb 25;14:634784. doi: 10.3389/fnmol.2021.634784 (PMC7947810; doi:10.3389/fnmol.2021.634784)
Supplement: Supplementary file 1 [file Data_Sheet_1.PDF]

Table S1

Table 1: Potential CG5003 effectors identified by mass spec analysis

Upregulated proteins	
protein name	CG Number
Zasp52	CG30084
Cpr30F	CG31876
	CG4588
Cyp313a1-RA	CG3360
	CG8993
Nplp3	CG13061
Downregulated proteins	
protein name	CG Number
Nep2	CG9761
GstD3	CG4381
Cpr47Ee	CG13222
	CG17111
Cpr47Ee	CG13222
b	CG7811
	CG7219
	CG9775
SC35	CG5442
Glt	CG9280
vnc	CG11989
	CG34325
Rpi	CG30410
Mp20	CG4696
	CG7203

Proteins are listed in the descending order of significance values.

Figure S1

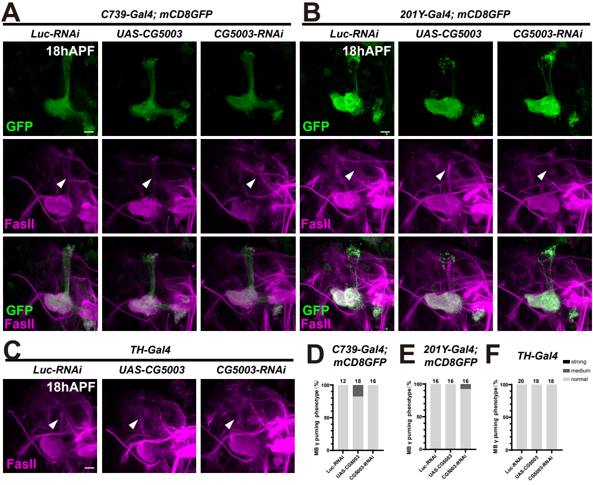

Figure S1. CG5003 expression in subsets of MB neurons or DA neurons does not affect γ axon pruning (A-F) Representative confocal images (A-C) and quantifications (D-F) of MB γ lobe formation of control, *CG5003*, and *CG5003-RNAi* fly pupae at 18h APF. *CG5003* or *CG5003-RNAi* was expressed in α/β (*C739-Gal4*), γ (*201Y-Gal4*), or DA (*TH-Gal4*) neurons. Note that FasII-positive γ lobes remain eliminated in all conditions. White arrowheads indicate the vertical α lobes. The N number of brains dissected and quantified for each genotype is indicated on the Figure. Scale bar: 50 µm.

Figure S2

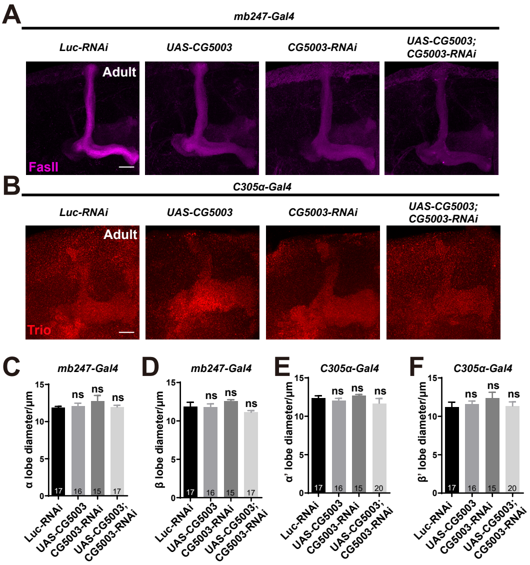

Figure S2. α/β lobes remain normal when altering CG5003 expression in subsets of MB neurons (A-F) Representative confocal images (A and B) and quantifications (C-F) of MB α/β or α'/β' lobe formation of control and experimental fly brains at adult stage. Note that FasII-positive α/β lobes and Trio-positive α'/β' lobes remain intact when CG5003 or CG5003-RNAi is expressed in MB α/β or α'/β' neurons. Average 15 brains (15 α/β lobes or α'/β' lobes) for each genotype were dissected and quantified. Scale bar: 50 μm.

Figure S3

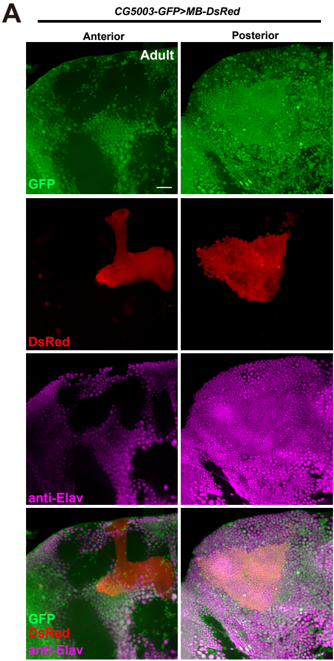

Figure S3. CG5003 is expressed in neurons Anterior and posterior part of the adult brains carrying the *CG5003* promoter-GFP and *mb247* promoter-dsRed (labeling MB) were immunostained with the anti-Elav antibodies. Note that GFP-positive signals are detected in the Elav-positive nuclei predominantly around the MB calyces, whereas some GFP-positive signals are also seen within the MB calyces.
